# The Contagious Diseases of Domestic Animals§Paper read before the American Health Association, Savannah, November 29, by Ezra M. Hunt, m.d., Secretary of State Board of Health, N. J.

**Published:** 1882-07

**Authors:** Ezra M. Hunt


### THE CONTAGIOUS DISEASES OF DOMESTIC ANIMALS.§ By EZRA M. HUNT, M.D.

The degree to which the diseases of animals are now attracting public attention has many foundations of propriety and necessity. If it were only that their sufferings and their wants appeal to our sympathy, and have claims upon our care, that appeal ought to secure some intelligent attention to their ailments. But they have become more intimately associated with us, in a scientific and practical sense, by the revelations and advancements of modern science. Biology compels us to include all life, and all its limitations, in our studies. Physiology and pathology, as applied to the whole animal world, indicate that the old recognitions of a comparative anatomy were but feeble expressions of those laws of symmetry which, in similarities and dissimilarities, in health and in disease, make the comparative study in physiology and pathology of still broader import.

---

§ Paper read before the American Health Association, Savannah, November 29, by Ezra M. Hunt, M.D., Secretary of State Board of Health, N. J.

Professor Flowers, in his address at the opening of the anatomical section of the late Congress, speaks of the "great revolutionary wave which has changed the whole aspect of biological ideas, borne onward mainly by the enormous advances in our knowledge of zoölogy, comparative anatomy and embryology."

Comparative anatomy was sure to become a prominent study so soon as we learned that the study of function is fully as important as that of structure.

Buckle, in his History of Clvilization, says: "The best phys--ologists distinctly recognize that the basis of their science must include not only the animals below man, but also the entire vegetable kingdom, and that, without this commanding survey of the whole realm of organic nature, we cannot possibly understand human physiology; still less general physiology."

It is inevitable that comparative pathology must embrace all the diseases of animal and vegetable life. Sir James Paget, in his address, some months since, on elemental pathology, traced the comparative diseases of plants and trees, and morbid growths thereupon, in a way that showed how in this sphere we have much to learn. Dr. Wilkes well says the comparison of disease in men and animals may throw much light upon its nature; and it is remarkable that so few persons have been stimulated to the work by considering the long controversy which has taken place as to the relation between vaccinia and variola, or hydrophobia and rabies. A true human pathology should have its basis in comparative pathology. Here lies a "mine of wealth but little worked." It is evident that in all these directions there are demands for the closest study and comparison.

But to the disciples of prevention and the health publicist there are inducements and obligations additional to these. Many of the diseases which it is our duty to prevent are derived or derivable from animals.

The list furnished by Professor Law to the National Board of Health names ten contagia and twenty-two parasites common to men and animals, besides fifteen parasites causing enzoötics in animals.

Dr. Charles Cameron, chairman of the health section of the late English Social Congress, says: "Recent discoveries show the enormous importance of the study of comparative medicine and pathology. It is only by such knowledge that preventive medicine can be most rapidly and surely advanced."

There is not merely the incidental fact enlarged upon by Ernest Hart, when he tabulates fifty typhoid epidemics, fourteen of scarlatina and seven of diphtheria, as somehow having their origin through milk supply.

Tuberculous disease is now claimed to be transferred by milk from animals to man, as well as the foot and mouth disease so much dreaded in England. Forms of anthrax and splenic fever and erysipelas, and perhaps some forms of boil, carbuncle and skin diseases, depend on specific conditions of diseased meat.

Some allegations have also been made as to ill effects from the flesh of so-called hog cholera disease, and as to lard made into butterine therefrom.

It becomes our duty to prevent disease in the lower animals, if only to prevent their transfer to mankind. The prominence which the study of parasitic diseases has very properly assumed attaches to this study a vast importance.

Dr. Cobbold, the eminent helmintologist, has catalogued upwards of 200 specimens in the museum of the College of Surgeons, London, in order "to show every parasitic animal that can affect the human body, and a selection of the principal types of those that inhabit the lower animals, especially such species as are associated with man."

To these we are constantly having additions, as in the new nematode discovered by Bastian, in the case of the boy who was at first supposed to have died of typhoid fever on the training ship Cornwall.

So impressed have not only professional men, but the public, become with the liabilities to various diseases that may occur through the flesh or milk of animals, that there is still another view that presses itself upon our attention.

There is no class of ailments against which the individual is so helpless to defend himself, and hence governments are striving to find out the extent of such maladies, and the means for their limitation and extinction.

Already, as we are well aware, the subject comes up as a great commercial question, and as involving great economic questions as to food supply.

No one can witness the rigid inspection now being had in England of all foreign cattle and sheep, and the interdiction which has so fallen upon American swine as almost to banish its live-stock from foreign markets, and to affect the trade in hams and salted pork, without realizing that the question has assumed importance which, alike in the interests of health and of trade, deserves the closest attention of all sanitarians.

Great Britain imports 5,250,000 cwt. annually.

In the present paper we propose to refer to most of these various diseases with only brief allusions.

Foot and mouth disease has, fortunately, not planted itself upon American soil. In the cases alleged to have come from our ports, Dr. Lyman, the chief veterinarian of the Agricultural Department, has been able to show that they were not American cases, but two bulls and eight heifers sent from England. They were promptly quarantined, and no spread among our cattle occurred. Yet because of the degree to which this disease, if imported, would affect our meat, and still more our milk supply, it is incumbent upon us to take all proper means to secure its exclusion.

Contagious pleuro-pneumonia, although occasioning great loss of stock and milk supply, has not as yet been proven to cause any specific disease through the meat or milk. Yet the trend of present opinion (see Cameron, etc.) is to look with suspicion upon it, as well as to regard it as a threatening source of inferior meat supply. There are urgent reasons why it should be watched and studied by sanitarians, both in the interests of health and of trade. For it is to be remembered that whatever reduces the quality of meat or adds to its scarcity, is at least indirectly the cause of ill health, since the poorer classes suffer physically from the higher prices, and have to feed on inferior flesh.

Splenic fever and other forms of anthracoid disease are of great interest to us, because they may be communicated by inoculation, or by the eaten meat, and also because the Texas cattle fever bears a suspicious relation thereto, so that it is called, by some good authorities, a splenic fever, or anthracoid disease.

While the most fatal and pronounced form of splenic fever is more common among foreign cattle than with us, these allied forms are to be closely studied, and guarded in the interests of public health. All cattle now going from America are closely watched for Texas fever. Recently we saw som cases in the yards at Birkenhead, which were feared to be of this or some splenic variety.

There is no contagion more dreaded than that of these anthracoid diseases.

In France the disease, as it affects sheep, is a loss, we are told by Pasteur, of twenty million of francs, or four million of dollars, annually.

Our losses by the Texas cattle disease have been large enough to lead to close inquiry, and the production or sale of such meat comes immediately under the commendation of the sanitarian.

Within the present month it has been my duty to exercise watchful oversight of a localized outbreak of anthrax. Ten cows, three horses, three sheep, one hog and several pigs have died, as if infused with a virulent poison, and post mortems have indicated the splenic character of the outbreak. The swine perished within forty-eight hours after having gotten a taste of one of the lungs, and a bull, which had broken into the pasturage, died in three days. A farmer who assisted in a post-mortem, and whose hand had no abraded surface, had swelling of the arm and other symptoms, which required medical care.

The swine disease, known as pneumo-entertis, swine plague or hog cholera, has prevailed in at least thirty of the States, and the losses by it have been enormous. The special report, No. 12, of the United States Department of Agriculture, 1879, refers to a tabular statement for 1877 of the losses of farm animals, chiefly from infectious and contagious diseases. The amount is stated at $16,653,428. About two-thirds of this sum was occasioned by the loss of swine by diseases presumed to be of an infectious or contagious character.

This particular disease continues to prevail in many parts of the country, and is far more common than in Great Britain or on the continent of Europe. Yet Fleming, in his *Veterinary Journal* for September, 1881, speaks of it as "an inoculable disease, due to a particular germ, which in Europe and America threatens to ultimately exterminate the porcine species."

Because it does not produce such a disease as trichinosis, its effects upon the quality of food supply must not be overlooked.

The immense losses not only affect the poorer classes by causing higher prices, which means a diminished supply of meat, but furnishes a great quantity of meat of inferior if not of diseased quality. There have been cases of sickness from pork that have not been caused by trichinæ. The new nematode found in the boy who was thought to have died of trichinæ on board the ship Cornwall (see report of Mr. Powers and Dr. Bastian, local government board, 1879), has been already alluded to.

Ballard and Klein have recently reported an acute specific disease produced by eating pork infected with a species of bacillus in two series of cases (see State Med. Sec., meeting Med. Congress, 1881), the pork being claimed to have come from America. The disease might be called a pneumo-enteritis, as both lesions were found.

Prof. Tidy, chemist of London Hospital, in some recent researches as to poisoning from eating pork sausages, thinks he has shown an alkaloid body and some chemical changes to have caused the sickness. Meat of diseased animals putrefies more readily and shows a tendency to a septic condition which the juices of the stomach are not always able to resist.

Cases of meat poisoning that are not specific in any known sense, are multiplying.

There is a very great temptation in the case of small animals, like swine, when a disease breaks out among them, to hasten them into the potted meat can, the sausage machine, or the salt pork barrel. Any disease so extensive in its ravages and so deadly in its type, must be carefully studied by the sanitarian as a guardian of the public health.

To the disease known as trichinosis we need to turn especial attention, because of its direful results, its real existence, and the interdiction it has caused to our own great pork trade in almost every foreign country.

It is not necessary here that we sift all the historical and political facts which have to do with this trade interruption. Enough is real to justify serious attention, and enough false or accepted on insufficient evidence to astonish us that countries which have long been the recognized producers of cases of the disease should all at once have seemed to assume that trichinosed pork is an American production. Neither is there need that we fully trace the disease and its results, since this has been so often and daily done, and because the monograph by our fellow member, the lamented Surgeon W. C. W. Glazier, of the Hospital Marine Service, has so fully presented it.

We think he and others show that its occurrence here in proportion to the number of our people and our immense number of swine is not equal to that of other countries. This nematode was first discovered in Guy's Hospital, London, in 1833, and up to 1836, inclusive, nine cases had been found among hospital patients in that city; one in Bristol, England; and six in Ireland. In 1843 the trichinæ was observed in Denmark, and not long after in Germany. It is to be remembered that all these cases, as also the monograph of Luckhart and the paper of Zenker, as well as most of the observations of Virchow, antedate the record of any fatal case in the United States. The first series of American cases are those alluded to by Virchow, as having occurred in Davenport, Iowa, in 1856, in a German family. The mother returned to Germany in 1861 and died in 1864 in a German hospital.

In her breast, which had been extirpated for cancer, encapsulated trichinæ were found. Before this the disease had become well-known in Great Britain and Europe, and perchance the German woman was a host of the cyst on her first arrival here.

The report of Dr. J. L. W. Thudicum on the parasitic diseases of quadrupeds used for food, is the report of the medical officer of the Privy Council, 1864, covers the history up to that time. The list of Dr. Glazier of trichinosis epidemics in Europe since 1860 (pp. 82–87), and that of cases in the United States reaching to 1880 (p. 172), completes the record, and shows how great a portion of the aggregate has occurred in other countries. His list of epidemics in Europe since 1860 comprises "about 160 epidemics, with 3044 cases, and 17 places where no number is given." Allowing, according to Meissner three cases each for places where no number is given, the whole numbers will reach 3095, with 231 deaths, making a total of about 150 epidemics, 3800 cases, 281 deaths, and 700 cases and 50 deaths not in the list. See p. 87, Glazier.

A similar record as to the United States up to 1880, inclusive, gives 26 localized epidemics, with 77 cases (a few cases number not known) and 26 deaths. While neither reckoning shows absolutely the relative degree of the disease, yet it does indicate that we are to look abroad for its greatest prevalence. But, as it appears "that only one-fourth of those eating the flesh from a trichinosed hog are affected with trichinosis," and, as Pagenstecher says, "five per cent. of the cadavers in Berlin hospitals contain trichinæ," and as the disease is well proven to exist here, we cannot be too diligent in preventing it from becoming as prevalent here as it is in foreign countries. All the more because of our immense production, and of the demands of home and foreign health. The last census gives the aggregate of swine at 37,396,621, which includes only those kept by persons having land.

I am aware that large statements come to us (such as one, not long since, from Lyons, in France, and another from Boston, in the United States), which would indicate a much greater prevalence of trichinosed pork than is generally supposed.

This might occur from the observer having lighted upon a special run of the disease.

But I have also learned that the observation of microscopists, like those of clinicians, are not always correct; that their experiments, like our experience, although honest, may have sources of error, and that the cautions given by Surgeon Sternberg, on page 5 of his excellent preface to his "Translation of Magnin's Brochure on the Bacteria," are to be observed as closely as what is seen under the microscope.

The French decrees of February last, the action of the English government, and that of some States on the continent, led our own government to an investigation which needs to be more exact and extended, but which, nevertheless, shows that the prohibition of importation was not well advised.

While there is still a kind of restrictive legislation which is unwise, yet we believe there is more of a disposition to get at the precise facts in a more authentic way, and to regulate commerce on the basis thereof. In this connection I submit a brief extract from a debate in the House of Lords, on May 16, of the present year, a summary in the New York *Times* of September 14, 1881, based on the testimony of Consul Mason, of Basle, to our government, and a communication from Dr. Lyman, Veterinarian of the United States Department of Agriculture, of date October 29, 1881, in answer to certain questions I had asked.

(*a*). In the House of Lords, May 16, of the present year, Lord Stanley, of Alderly, asked Her Majesty's Government whether they would prohibit the importations (alluding to pork), of which a large proportion were infected with trichinæ and bacilli; also of such oleomargerine or butterine as contained the grease of pigs suffering from the new disease of swine at Chicago, and the fat of horses infected with disease or such as was adulterated with soapstone. The Marquis of Huntley said he could assure the noble lord that there was no established case of American hams being infected with trichinæ, and there had been one case only by infection by bacilli. (See Fleming *Vet. Journal*, June, 1881, p. 462).

Consul Mason writes from Basle that abundant and unmistakable evidence shows that the panic about American meats has begun to decline. From the first it was artificially created, mainly by such official action as the French decree in February prohibiting importation of American pork, and a published document from the Minister of the Interior in Neufchatel, asserting that the microscope had shown more than half of American hams and canned meats to be infected. That canton takes most of its 150,000,000f. of exports from this country in the form of meats, and the trade has been almost destroyed for the present, although no case of trichinosis has ever been reported there. But as consumers, who have taken no part in the scare, have found themselves suffering from advanced prices, they have begun to complain, and as the sales of domestic meats, in the difficulty of discrimination, have been affected, dealers who thought they saw "protection" in the scare have joined the demand for removal of the prohibition. The leading meat importer in Basle has for several years subjected all his meats, from whatever country, to microscopic examination by the official city inspector; the occasional pieces containing traces of defunct trichinæ were saved, and were experimentally fed to hogs, chickens, cats and dogs, but at the end of six months no trace of disease could be found in the flesh of these animals. The "syndicate of lard and salted meats" of Bordeaux made an elaborate protest against the French decree, saying that in twenty-five years only a single case of the disease had occurred in France, and the character of that was disputed; that Germany has excluded only chopped meat for sausages; that there is no prohibition in Europe, except in Italy, and perhaps in Spain; that no decrees can keep out American meats, although disguise may be forced; and that it is supreme folly to injure the people of France for the sake of an imaginary danger. The Swiss *Journal de Geneve* remarks that the Ministers in Paris, London and Brussels have been compelled to acknowledge that not a case of trichinosis from American meats has ever been authoritatively shown to have occurred in Belgium, France or England, and it ascribes the scare to the jealousy of European meat growers and the desire of speculators in America to break the pork market, saying that a single Chicago firm is reported to have cleared 30,000,000 francs in one year by a skillful combination. Mr. Mason thinks the foreign trade in salted meats can never be fully restored without establishing in this country a system of official inspection which shall carry with it the weight of State or Federal authority. German municipal experience, notably in Berlin, has shown that a capable inspector can examine several hundred pieces per day, and he thinks young men of ordinary education and good eyesight could be trained in a fortnight, by a competent microscopist, to do this work, and that the cost need not exceed three cents per hog. Until this or some equivalent step is taken, the one per cent. of diseased hogs admitted to exist here will be a serious obstacle to the export trade in meats, for European meats are officially inspected, and to be able to say that American are not, is to give opponents of our trade the power to keep up a prejudice. An official report in review of the whole subject is needed, showing by statistics the extent of the hog product in this country, the immunity of our people from disease, how many hogs die of cholera and transportation accidents, and what becomes of them, and the actual values of land and corn in the pork-growing States. It is often published, and is more or less believed, that the comparative cheapness of our meats abroad is because the bad or questionable qualities are exported. This can be combated by showing that the cost of growing the best quality here permits exporting it at the prices it bears abroad. Such a report should be published under an official seal, which is, of all things, the most authoritative with a European, and should be distributed abroad.

DEPARTMENT OF AGRICULTURE,
WASHINGTON, D. C., October 29, 1881.

*E. M. Hunt, M.D., Secretary, etc., State Board of Health, Trenton, N. J.,*

MY DEAR DOCTOR:—Your letter of inquiry of the 22d inst. is duly received. I will answer your questions as fully as I am able to do so. You ask:

1. State the comparative number of cases of trichinæ that have been identified as to their origin, viz.: how many American, English, French, Continental?

If this refers, as I suppose it does, to disease in humans, I am unable to answer.

2. How many live hogs are claimed to have come from America with trichinæ?

From the annual report of the English Veterinary Department for 1879 I copy the following:

"The slaughter of large numbers of American swine at the port of landing afforded opportunity of obtaining specimens of flesh for examination, with the view to ascertain what proportion of the animals were infected with trichinæ. The inspectors of the Veterinary Department examined two hundred and seventy-nine separate portions of swine's flesh which were sent from Liverpool, and detected living trichinæ in three specimens. * * * No doubt, therefore, existed as to the dangerous character of American pork, and a consultation on the subject took place with the medical officers of the Local Government Board. The matter was also discussed in the House of Commons; but it was not deemed expedient to prohibit the introduction of American pork into this country, for the reason that such a measure would have damaged the trade without producing any satisfactory results; * * * besides which, trichinosis among swine is known to exist in Germany, and it probably exists in other exporting countries, so that nothing short of total prohibition of swine flesh in all forms, from all sources, would have been effectual. The possibility of our own swine being to some extent infected with trichinæ has been suggested; the result, however, of many examinations has, up to this time, been negative."

I do not know that Germany or France has even examined for this disease in live hogs.

3. How much shipped pork has been identified with it?

To foreign countries, Germany reports each year some, if I remember rightly, three or four thousand "pieces" of American pork. Where they get them, what a piece is, or what percentage of pieces examined and found diseased, I have never heard of their saying. I do not know that they have made any publicly, neither do I know that other foreign countries have done any more than to say that they found, upon examination, that trichinæ existed in American pork, as they had seen it arriving in their countries.

4. Have Western hogs in any one section been more affected?

It has no such favorite locality that we know of, although this will be a good ground for future investigation.

5. Are trichinæ found in the fat, as in cold tried lard?

I have until now thought not. Professor Taylor, of this department (microscopist), tells me that in the journal of the Microscopical Association he has recently seen that they have been found in fat. I should rather see than believe without so doing. A veterinarian, of New England, informed me on April 14 last, that he had examined portions from 2,701 Western hogs, obtained in Boston, 154 of which he found infected—*i. e.*, one case to each 17 54–100 hogs examined. He tells me that he will make a statement to this Savannah meeting, that he has examined portions of 8,773 Western animals, and has found one case to every twenty five animals. You will see that there is a great difference between his first (April) examination and this one, and his result is so greatly different from the English examination of our hogs, above mentioned, and so much above any known percentage among animals of every other country, that I cannot but entertain doubts of the value of his examination.

While such facts are to be borne in mind, the gravity of the disease, its cosmopolitan character, and the interest both of health, life and trade, require that, under the head and authority of the United States government, there should be such testing of facts as will be fully reliable, and not depend on single observers, and that some plan may be devised to secure both to animals and mankind immunity from this direful invasion.

The latest and most graphic outline of the disease is given incidentally in a scientific address by the veteran naturalist, Richard Owen, who gave it the name trichinæ. It is worthy of transcription: "The finding of the wormlet within the cysticule in 1835 (proceedings of the Zoölogical Society of London, February 21, 1835; also Transactions, ibid. 4 to Vol. I), and the more important discovery of Dr. Zenker, of Dresden, in 1860, of its causative relations to a direful disease, have demonstrated that the several groups of symptoms to which I have referred under the respective technical denominations applied to their groups, may, one and all, be due to the deglutition of trichinæ spiralis."

It is thus all the more serious, because the symptoms simulate so many diseases, such as dyspepsia, common diarrhœa, dysentery, peritonitis, pneumonia, rheumatism, gout, typhoid fever and endocarditis. The larvæ of this wormlet in the flesh of the animal it infested, being introduced as food into the human stomach, finds in that warm cavity an environment of mucochymous nutriment, in which it rapidly matures, acquires activities, and develops its generative organs and products. If permitted to pass into the intestinal canal, it there excludes its progeny, which also rapidly acquire their full size and procreative faculty. But the grave symptoms of their presence are due to the curious migratory instincts of the young trichinæ, which impels them to make their escape by perforating the tunic of the intestines, in the course of which operation the majority find their way into the venous capillaries. Such as wriggle through the meshes of the vascular network, bore their way through the serous tunic of the gut and pass into the abdominal cavity, whereupon the peritonitic are complicated with the enteritic symptoms. But the majority are carried to the right side of the heart, and thence by the pulmonary artery to the lungs. Treading then the capillaries continuous with the commencement of the pulmonary veins, the trichinæ are brought back to the heart. As many as may have burrowed into the vascular walls of the right or left ventricle, or may have got into the cavity of the pericardium, give rise to the symptoms summed up in the terms of art already cited.

Trichinæ, which may have strayed into the tissue of the lungs, or which may have wriggled through the pulmonic serous covering, and from the pleural cavity may have invaded the serous membrane on their way to the intercostal muscles, add the pleuritic and pulmonic to the pericarditic symptoms. The natural affinity or attraction of the trichinæ is to myonine or the muscular tissue. There their wanderings come to an end. They are conveyed so soon and so rapidly by branches of the carotids to the muscles of the larynx that the trichinæ are there found most constantly and abundantly, but usually so vast is their number that they are carried to the voluntary muscles of the entire body. In the exceedingly delicate connective tissue of the ultimate bundles of the ultimate fibrils, the young trichinæ coil themselves up to their larval repose, exciting no other organic change than an overflow of plasma, which condenses with the contiguous cellulosity and becomes molded into the shape of an elliptic case, in which may be seen under the microscopic compressorium one or more of the tiny worms disposed in two or more circular coils.

An ounce of hog's muscle has been found to contain 85,000 trichinæ, while Thudicum estimated 40,000,000 in the body of a German who died in the London Hospital, and Cobbold, who reviewed the calculation, thought this below the number. He calculated in the flesh of a hog, that had caused an epidemic in England, 80,000,000. Thus far the record of cases in Germany and of endemics thereof has been ahead of that of any other country.

The number of cases recorded by Glazier for the United States, from 1864 to 1880, is seventy-eight, while post-mortems have afforded proof of their existence many times.

We believe there is nothing to point to this disease as more prevalent in the United States than in other countries. Yet it is prevalent enough, and its occurrence possible and serious enough to demand the closest attention of all those whose effort it is to prevent disease.

The immense swine production and pork traffic of this country, of course, makes the aggregate number of cases larger, and at the same time, enforces the interest and the duty of leaving no measures that are expedient untried to limit or destroy the disease. Not because it is an American parasite, for Heller (Ziemssen Enc. III., p. 628) says that trichinæ have been found in all countries where search has been made for them, and Pagenstetcher says: "They are cosmopolitan with man, hogs, rats and mice."

As it is not like a disease spread through the air, and does not rapidly pass from one animal to another, save where there is a source of production, there are great encouragements for an attempt to eradicate the disease. There is some reason to believe that rats frequenting hog pens are often its conveyancers, and that we need some "Pied Piper of Hamelin" to drive them all into the sea. Cobbold says infection must be due to the facilities for swallowing garbage, especially dead rats. It is an established fact of natural history that hogs are not by nature filthy in their habits or in their choice of food. If mankind thrust upon them all sorts of improper food, all the indignation must not be projected against the brute. A treatise on the hygienic management of swine is in order, especially as they have become subjected to such invasions.

All serious diseases which are either directly transmissable to man, or which by the diseased or imperfect meat and milk they put on the market, or by the actual loss of food supply they occasion, must attract the attention of all sanitarians.

And as our foreign trade is being so much affected, both in the interests of health and of commerce, we should join in consultation and advice with those who have to guard our commercial and national interests.

We may call it prejudice or retaliation, and to some extent correct the distorted views of some of our British and continental friends, but so long as there is a basis of truth it behooves us, both in our home and foreign interests, to do all that is intelligent and practicable in stamping out or limiting these diseases.

For this we have a few suggestions to make:

1. There is a need of accurate study into causes as well as into methods of limitation. Our government has done some good work in the way of investigation, and the present distinguished head of the Agricultural Department, Dr. Loring, is by profession, agricultural knowledge and executive experience, well fitted to do more.

There are some veterinarians that are at work, but the number of these is small, and it is difficult to find enough others who are capable.

We feel that medical men and others of this association should feel these investigations to be as worthy of their labor as those directed to some human diseases. The time has come when medical and sanitary experts should recognize the study of the comparative plagues as a part of their work and devote the closest study thereto.

2. Some form of direct oversight should be exercised in every State over the communicable disease of animals. There is no more economical outlay than that which guards our milk and meat supply. An authority in every State to oversee the work and to circulate information among farmers and stock raisers, to obtain knowledge through local township committees, supervisors or health boards of special outbreaks and to secure from assessors exact statements each year of all deaths and known causes of deaths of animals would be of essential service. Thus infected districts would soon be singled out and the disease ere long eradicated. In Germany microscopical examination of pork has been made compulsory.

3. Public abattoirs and inspection thereat should be encouraged.

4. All live stock sent out of the country should be inspected at the point of final shipment by a government veterinary inspector.

5. Pork packing and smoking establishments should guarantee their methods, and be subject to penalty if the meats furnished by them are proven to have caused disease. Trade marks and brands are easily made, and severe penalties to producers for any meat that is proved to have caused sickness is one of the best precautions. It will then be the interest of large establishments to protect themselves by sampling lots with the microscope and by other expert examinations.

6. A system of inspection is desirable; but we do not wholly rely upon it, as being exceedingly expensive, and as not alone accomplishing as much as when joined with other methods.

7. All wholesale lots of chopped meats or sausage or of potted meats should be certified by competent meat inspectors.

8. It should be known that thorough salting and cooking remove all risks from animal parasites. Virchow, in treating of this disease, expresses his regret that the old forms of curing and smoking have had so many substitutes.

9. Careful inquiries should be made as to the presence of parasites in fats, and as to the fats of diseased meats, lest lard and oleomargerine, etc., may be so prepared as not to be subjected to proper heat, and our risks be increased in this direction.

In view, therefore, of the vast interests involved to health, not less than to commerce, we trust this association will direct still more careful attention to the communicable diseases of animals, and seek to find their causes, and arrest their courses, since, be it man or beast, it is a common concern in a common welfare.

We append the approximate summary of the last census of all live stock in the States and Territories, as found on farms or with those owning land. The aggregate shows a yearly product of one hundred million, if we estimate all, used as food:

### FOOD STOCK IN THE UNITED STATES.

| STATES AND TERRITORIES. | Working Oxen. | Milch Cows. | Other Cattle. | Sheep. | Swine. |
|---|---|---|---|---|---|
| Alabama | 75,534 | 271,443 | 404,213 | 521,267 | 1,252,462 |
| Arizona | 984 | 9,156 | 34,843 | 112,107 | 3,819 |
| Arkansas | 25,444 | 249.407 | 433,392 | 375,795 | 1,565,098 |
| California | 2,288 | 210,078 | 451,941 | 5,644,341 | 603,339 |
| Colorado | 8,179 | 28,770 | 315,989 | 994,844 | 7,656 |
| Connecticut | 28,399 | 116,198 | 92,121 | 104,592 | 63,662 |
| Dakota | 11,418 | 40,572 | 88,825 | 47,116 | 63,394 |
| Delaware | 5,818 | 27,284 | 20,450 | 40,044 | 48,186 |
| District of Columbia | 4 | 1,292 | 271 | ............ | 1,032 |
| Florida | 16,141 | 30,359 | 414,869 | 80,169 | 287,051 |
| Georgia | 50,026 | 315,073 | 544,812 | 752,490 | 1,471,003 |
| Idaho | 737 | 12,838 | 71,292 | 39,246 | 14,178 |
| Illinois | 3,346 | 865,913 | 1,515,093 | 1,544,728 | 5,170,1[illegible]8 |
| Indiana | 3,970 | 504,944 | 865,136 | 1,614,073 | 3,186.413 |
| Iowa | 2,504 | 854,097 | 1,754,341 | 669,510 | 6,034,316 |
| Kansas | 16,789 | 418,333 | 1,015,935 | 733,275 | 1,787,969 |
| Kentucky | 36,076 | 301,882 | 494,743 | 1,678,515 | 2,225,225 |
| Louisiana | 41,729 | 146,454 | 282,418 | 203,177 | 633,489 |
| Maine | 43,049 | 150,845 | 140,527 | 944,856 | 74,269 |
| Maryland | 22,246 | 122,907 | 117,387 | 306,960 | 335,408 |
| Massachusetts | 14,571 | 150,505 | 96,045 | 109,796 | 80,123 |
| Michigan | 39,493 | 384,573 | 467,060 | 3,065,533 | 964,071 |
| Missouri | 9,020 | 661,405 | 1,410,487 | 2,193,006 | 4,573,123 |
| Montana | 936 | 11,308 | 160,143 | 211,225 | 10,278 |
| Nebraska | 7,234 | 161,609 | 613,129 | 285,935 | 1,241,724 |
| Nevada | 765 | 13,319 | 158,137 | 193,894 | 9,080 |
| New Jersey | 2,022 | 152,078 | 69,786 | 237,368 | 219,069 |
| New York | 39,633 | 1,437,855 | 852,283 | 2,718,093 | 751,916 |
| North Caroliua | 50,188 | 232,133 | 375,105 | 690,894 | 1,453,541 |
| Tennessee | 27,337 | 303,832 | 444,736 | 1,079,743 | 2,158,169 |
| Virginia | 54,709 | 243,061 | 388,414 | 823,776 | 956,451 |
| Wisconsin | 28,762 | 478,374 | 622,005 | 1,921,047 | 1,128,239 |
| | 671,351 | 8,897,917 | 14,811,928 | 30,027,415 | 37,369,621 |

### RECAPITULATION.

| | |
|---|---|
| Oxen | 671,351 |
| Milch Cows | 8,897,917 |
| Other Cattle | 14,811,928 |
| Sheep | 30,027,415 |
| Swine | 37, 396,621 |
| | 91,805,232 |

—*Journal of Comp. Med. and Surgery.*

